# Pioneers and paradigms in sprint science: a thematic historical mini review

**DOI:** 10.3389/fspor.2026.1843352

**Published:** 2026-05-18

**Authors:** Jiahua Li, Xiaoge Xiao, Yifang Fan

**Affiliations:** School of Running, Fujian Normal University, Fuzhou, China

**Keywords:** biomechanics, exercise physiology, sprint performance, sprint science, sprint training

## Abstract

This mini review traces the historical evolution of sprint science by examining the pivotal contributions of key figures across interconnected disciplines. Focusing on thematic developments, this paper investigates how physiological foundations, biomechanical analysis, strength theory, quantitative approaches, and applied coaching practice have collectively shaped the discipline. Specifically, this synthesis highlights the physiological bioenergetics mapped by Archibald Vivian Hill, the foundational coaching methodologies of Sam Mussabini and Clyde Hart, the kinematic and biomechanical innovations of Ralph Mann and Richard Neil Hinrichs, the quantitative strength theories of Vladimir Mikhailovich Zatsiorsky, and the ground reaction force paradigms established by Peter Weyand. By chronologically synthesizing these advancements, the review illustrates that the maturation of sprint science did not follow a singular linear trajectory. Instead, the field emerged through the complex, synergistic integration of diverse knowledge domains. Ultimately, this synthesis suggests that future advancements in sprint science will depend upon the continued cross-pollination of these disciplines, particularly through the potential adoption of AI-driven models of data analysis to optimize performance outcomes and establish a more rigorous, evidence-based scientific framework.

## 1 Introduction

Sprinting is a widely studied event in sport because performance emerges from the interaction of physiology, biomechanics, technique, training practice, and other factors (1). Although sprint performance is often examined in relation to training status, physical capacities, and competition preparation (2), advances in sprint performance have also depended on the progressive development of scientific knowledge (3). Over more than a century, researchers have moved from describing the physiological basis of muscular work to analyzing sprinting as a complex mechanical system governed by force production, force application, and movement coordination, among other factors (4–8). Accordingly, sprint performance is best understood as a multifactorial outcome rather than as the product of any single physiological, biomechanical, or technical determinant (1, 9).

A useful starting point for this scientific trajectory is the work of Archibald Vivian Hill, whose contributions helped establish exercise physiology as a rigorous scientific field (6, 10). Hill's research on muscle energetics, heat production, oxygen uptake, and the force-velocity relationship provided a conceptual foundation for later work on human locomotion and athletic performance (6). Hill also examined athletic records through a physiological lens, using performance data to discuss the limits of oxygen uptake and the energetic basis of exercise, thereby representing an early attempt to interpret sporting performance with scientific analysis (10, 11). At the same time, sprint science also developed through the gradual systematization of coaching practice. In this respect, Sam Mussabini and later Clyde Hart helped move sprint preparation from experience-based technical guidance toward more structured and analytically organized training (12, 13).

As sprint research developed, the field gradually expanded beyond physiological description toward biomechanical explanation (14). This shift led sprint research to place greater emphasis on whole-body coordination, limb function, and the mechanical principles underlying force generation and transmission (14–16). However, early research focused primarily on the contribution of the lower limbs to sprint performance, particularly in relation to lower-limb explosive power, stride characteristics, and ground reaction forces (17–19). Against this background, Richard Neal Hinrichs' work on upper-extremity function showed that arm action in running should not be understood as merely auxiliary, but as part of whole-body coordination involving angular momentum regulation and movement control, thereby broadening biomechanical understanding beyond the lower limbs alone (20, 21). Within this broader development, Ralph Mann helped bring biomechanical analysis closer to coaching practice by showing that sprint technique and performance could be interpreted through measurable mechanical variables rather than observation alone (22). A related broadening of biomechanical thought can also be seen in the work of Vladimir Mikhailovich Zatsiorsky, whose research was not devoted exclusively to sprinting, but his studies of human locomotion, three-dimensional biomechanical modeling, and movement mechanics contributed to a more systematic understanding of running as an integrated mechanical process (23, 24). Subsequent research in sprint mechanics has further suggested that sprinting should be understood not in terms of isolated body segments or single variables, but rather through the laws of motion and integrated models of step kinematics, contact dynamics, and center of mass behavior (7). In this respect, Zatsiorsky's work on full-body biomechanical analysis and the mechanical study of locomotion formed part of the broader intellectual background from which later running and sprint models emerged (23).

In recent decades, sprint science has become increasingly quantitative. Contemporary sprint science relies heavily on mathematical and biomechanical models not only to explain, but to accurately predict and optimize sprint performance (8, 25). These approaches include spring–mass-based models, force–velocity profiling, and other quantitative frameworks that have been used to analyze force application, stiffness, power, and spatiotemporal characteristics in sprinting, while also revealing the limitations of overly simplified mechanical interpretations (1, 5, 26). Regression models, differential equations, and related mathematical tools have become essential for interpreting sprint data, testing biomechanical hypotheses, and improving performance prediction (8). A particularly important development within this increasingly quantitative tradition was the work of Peter Weyand and his collaborators. Their studies challenged the long-standing assumption that faster sprinting is achieved primarily through more rapid limb movements, demonstrating instead that faster top running speeds are more closely associated with the application of greater support forces to the ground (27). Subsequent work also refined how these forces should be interpreted, including critiques of the passive, linear-spring stance mechanics assumed in overly simple spring-mass explanations, and the growing recognition that not only force magnitude, but also the timing, asymmetry, and mechanical effectiveness of force application, are central to sprint performance (7, 28, 29). Alongside these developments, strength theory also became increasingly relevant to sprint preparation. Here again, Zatsiorsky's influential work on task-specific strength, explosive strength, and the explosive strength deficit provided a theoretical framework for understanding how general strength may or may not transfer effectively to high-speed athletic actions such as sprinting (30).

Despite the richness of this literature, existing reviews have tended to examine sprint performance from relatively specific perspectives, such as elite sprint training, the biomechanics of the sprint start, or phase-specific biomechanical determinant (1, 15, 31, 32). As a result, less attention has been given to how these domains developed historically and how they interacted in shaping sprint science as an interdisciplinary field.

Against this background, this mini review traces the evolution of scientific contributions to sprint training and performance from early physiological foundations to contemporary biomechanical and mathematical approaches. Rather than treating sprint science as the product of a single discipline, this mini review examines how physiology, biomechanics, coaching practice, and quantitative modeling have interacted across time. Particular attention is given to the work of individuals who helped shape the field, including scholars whose work influenced sprint science indirectly through broader advances in biomechanics and strength training theory, and to the way mathematical methods have progressively strengthened explanation, measurement, and performance analysis in sprint research. Rather than offering another domain-specific summary, this review uses selected key figures as analytical anchors to examine the historical co-development of physiological, biomechanical, coaching, strength-related, and quantitative perspectives. Its contribution therefore lies not only in providing a historical overview, but also in clarifying the intellectual transitions and cross-domain interactions that underpinned the evolution of modern sprint science. This mini review aims to clarify how sprint science developed into an interdisciplinary body of knowledge and to identify the intellectual foundations that continue to optimize modern training and performance.

## 2 Methods

This study was designed as a narrative mini review with a historical and analytical focus. It was not intended to provide an exhaustive or systematic synthesis of the entire sprint-related literature. Instead, the review aimed to examine how selected scientists, scholars, and coaches contributed to major stages in the development of sprint science, from early physiological and coaching-based interpretations to later biomechanical and quantitative approaches.

Although no formal systematic review protocol was used, a structured narrative approach was adopted to improve transparency. Literature was identified through searches of major academic sources, including Google Scholar, PubMed, Web of Science, and relevant books and historical materials, using combinations of keywords such as “sprint performance,” “sprint training,” “sprint science,” “exercise physiology,” “biomechanics,” “ground reaction force,” “running mechanics,” “coaching,” and other related terms. Reference lists of influential publications were also examined to identify additional primary and secondary sources relevant to the historical development of sprint science. Priority was given to primary works and secondary sources that were historically influential, conceptually important, or frequently cited in later sprint-related research. Citation prominence was considered only as a supplementary indicator of influence rather than a stand-alone selection criterion. When broader thematic or field-based searches returned large numbers of publications, records were first screened by title and abstract, followed by full-text review of those judged to be directly relevant to sprint science and its historical development.

On the basis of this broader thematic screening, key figures were identified provisionally when their work appeared repeatedly in relation to major domains in sprint science or to important historical transitions in the field. The purpose was not to generate an exhaustive list of all contributors, but to identify analytically useful figures through whom major strands in the development of sprint science could be examined. After this initial identification, targeted author-based searches were conducted for shortlisted figures in order to verify their relevance to sprint science and to identify the publications most suitable for historical and thematic analysis. This additional step was used to capture not only widely recognized works, but also the broader conceptual and methodological contribution of each figure to the historical development of the field. An illustrative example of this author-based search and screening process, together with representative landmark publications for selected figures, is provided in the Supplementary Material.

The selection of key figures was based on three criteria. First, their work had to address a major domain of sprint knowledge, such as physiology, biomechanics, coaching methodology, strength theory, or quantitative analysis. Second, their contribution had to exert clear historical or conceptual influence on subsequent research or coaching practice. Third, sufficient documentary evidence needed to be available to support analysis of their contribution and significance. Applying these criteria after the broader thematic screening and the targeted author-based verification led to the inclusion of Hill, Mussabini, Hart, Mann, Hinrichs, Zatsiorsky, and Weyand were included as key figures for this mini review. The omission of other important contributors should not be interpreted as a denial of their significance; rather, the selected figures were chosen because they helped clarify major transitions and thematic strands in the historical evolution of sprint science.

This mini review combined thematic analysis with historical interpretation. Rather than just presenting the biography, it used the selected figures to examine several interrelated domains in sprint science, including physiological foundations, the systematization of coaching practice, biomechanical analysis, strength theory, and quantitative modeling. Attention was given to the historical context of each contribution, its principal concepts or methods, its influence on later sprint research and practice, and its limitations or boundaries within the broader development of the field. Table 1 summarizes the selected figures, their main areas of contribution, and significance for sprint science and practice.

**Table 1:** Key figures and their pivotal contributions to the multidisciplinary evolution of sprint science.

Key Figure	Primary domain/contributions	Significance to the evolution of the field	Impact on modern sprint science and practice	Limitations
Hill	Established key physiological concepts related to muscle energetics, oxygen uptake, oxygen debt, and the force–velocity relationship	Laid the physiological foundation for understanding muscular work, energy supply, and mechanical function in exercise, providing an important basis for later sprint research (6)	Retains relevance for contemporary understanding of sprint energetics, physiological limits, and the metabolic background of high-intensity performance	Hill's work provided a foundational physiological framework, but it did not offer a modern biomechanical explanation of sprinting. Some concepts, especially the classical oxygen debt model, were later revised or replaced by broader interpretations such as excess post-exercise oxygen consumption (33)
Mussabini	Combined systematic observation, technical analysis, individualized training design, and experiential coaching knowledge in sprint preparation	Represented an early bridge between empirical coaching practice and more systematic performance analysis, showing how technical observation and tacit expertise could be combined in sprint training (34)	Continues to influence the value placed on technical observation, individualized preparation, and coach-led practical judgment in sprint training	Mussabini's methods were historically important, but they remained grounded primarily in experiential judgment, observation, and tacit coaching knowledge rather than formal experimental science. His approach therefore lacked the measurement precision and analytical tools available in later sprint research (35)
Hart	Developed a highly structured approach to sprint coaching, especially in the 200 m and 400 m, with emphasis on training organization, athlete development, and relay preparation	Represented the further systematization of elite sprint coaching practice and demonstrated how long-term training design could be translated into consistent competitive success (12)	Remains influential in the structured organization, periodization, and event-specific management of sprint training, especially in the 200 m and 400 m	Hart's contribution lay mainly in the systematization of coaching practice rather than in formal laboratory research. Although highly influential in training design, his framework was less concerned with biomechanical quantification or mechanistic explanation than later scientific models of sprint performance
Mann	Integrated biomechanics into sprint and hurdle coaching through technique analysis, biomechanical performance descriptors, and feedback-based instruction	Helped bridge the gap between biomechanical analysis and coaching practice, influencing technical instruction in elite sprinting and hurdling (22)	Helped establish the modern application of biomechanical indicators, video analysis, and technique-based feedback in sprint coaching	Although Mann made an important contribution to translating biomechanics into coaching practice, sprint performance is a multifactorial outcome influenced by biomechanical, physiological, neuromuscular, anthropometric, and environmental factors (9)
Hinrichs	Demonstrated the biomechanical role of arm swing in angular momentum regulation, movement coordination, and stabilization during running	Expanded sprint biomechanics beyond lower-limb analysis and provided scientific support for the functional importance of upper-extremity action in running (36)	Supports contemporary whole-body interpretations of sprinting, especially regarding arm motion, coordination, and angular momentum control	Hinrichs significantly expanded understanding of upper-extremity function, but his contribution addressed one specific biomechanical dimension rather than providing a full general model of sprint performance. His work complemented, rather than replaced, broader lower-limb and force-based analyses of sprinting
Zatsiorsky	Advanced the biomechanical analysis of walking and running through three-dimensional full-body modeling, examined mechanical energy transfer in lower-limb movement, and developed influential strength-training concepts relevant to speed and power development	Although not exclusively a sprint specialist, he broadened the scientific basis of sprint research by linking locomotion biomechanics with strength and power training theory, thereby influencing both the analysis of running mechanics and the design of sprint-related explosive training (24)	Continues to shape sprint-related strength and power training through concepts of specificity, explosive strength, and transfer	Zatsiorsky was not a sprint specialist in the narrow sense, and many of his concepts were not developed specifically for sprinting. As a result, their relevance to sprint science is partly indirect and depends on later application and adaptation to sprint-related contexts (37)
Weyand	Showed that faster top running speeds are associated with greater ground forces and advanced quantitative, force-based approaches to the mechanical interpretation of sprint performance	Helped move sprint science toward force-based and mathematically grounded explanations of performance, contributing to later biomechanical research and interpretation (8)	Reinforced the modern emphasis on force-based interpretation, gait mechanics, and quantitative assessment in sprint performance analysis	Weyand's early force-based explanation was highly influential, but later studies, including subsequent work by Weyand and his collaborators, showed that this framework required further refinement through more explicit consideration of gait mechanics and their relationship to running ground reaction forces (38)

## 3 Thematic strands in the development of sprint science

**Figure 1:**
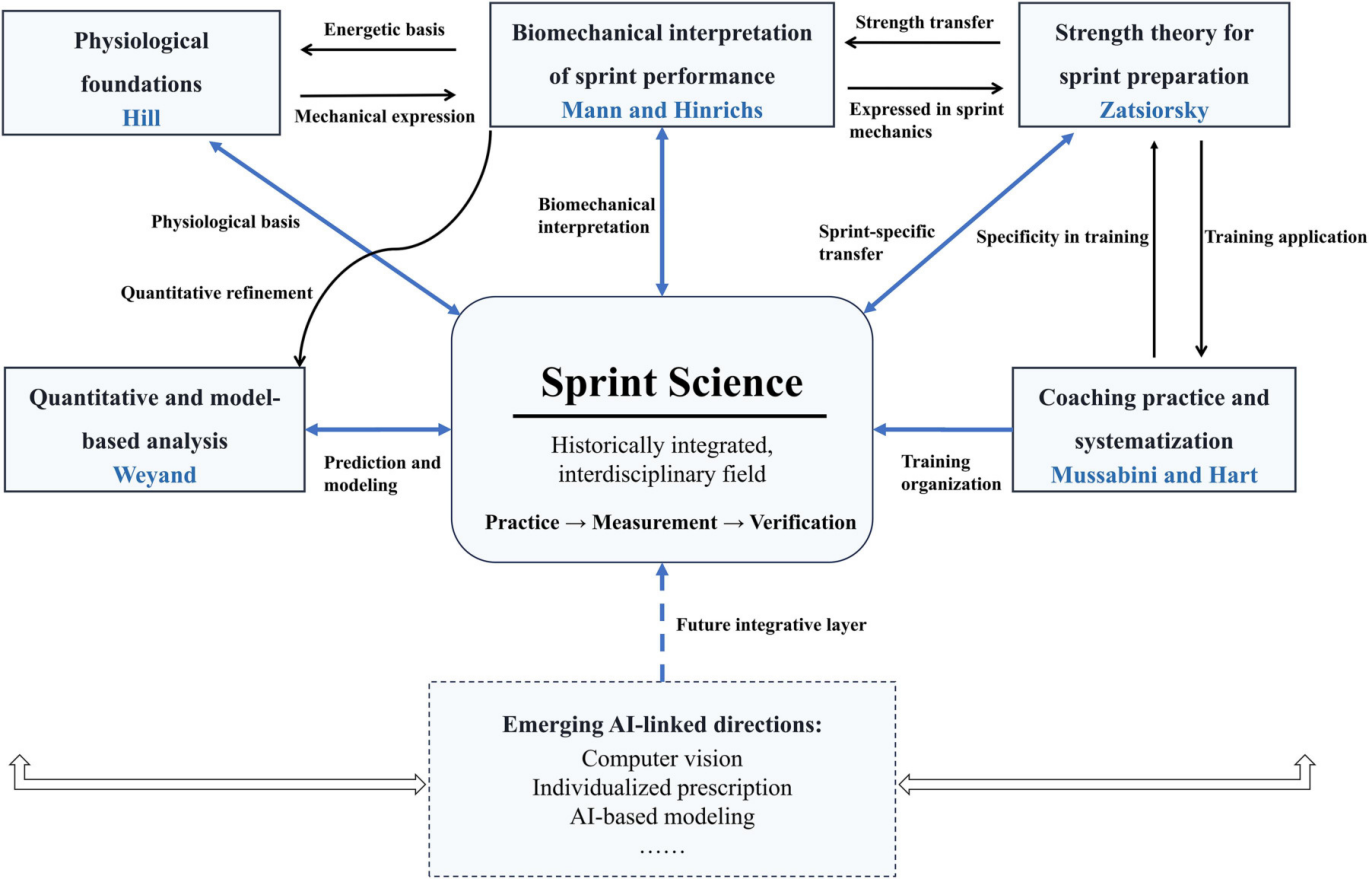
Conceptual strands in the development of sprint science.

The historical development of sprint science can be understood not only through individual scholars or chronological milestones, but also through several interrelated thematic strands that progressively shaped the field. These strands include the physiological foundations of muscular work, the gradual systematization of coaching practice, the biomechanical interpretation of sprint performance, the development of strength theory relevant to sprint preparation, and the later quantitative and model-based turn in sprint research. Although each strand had its own emphasis, they did not develop in isolation. Rather, they interacted with one another and collectively contributed to the emergence of sprint science as a more integrated and analytically grounded field. Figure 1 summarizes these thematic strands in the historical development of sprint science.

### 3.1 Physiological foundations

Hill is widely recognized as a foundational figure in exercise physiology (39). Hill's most important and lasting contributions were in muscle calorimetry, muscle mechanics, and applied exercise physiology, and his work helped establish physiological concepts that remain influential today; he was awarded the Nobel Prize in 1922 (6, 40). Although contemporary exercise physiology now extends across a much broader disciplinary landscape than in Hill's era, recent reviews continue to place Hill's work at the foundation of the field. In particular, Hill and Lupton's introduction of maximal oxygen uptake remains a central reference point, even though current interpretations of the factors limiting V˙O_2_max are more integrative than the original formulation (33, 41).

For sprint science, Hill's importance lies less in modern biomechanical analysis than in the physiological framework he provided for understanding muscular work and high-intensity exercise (11). Hill and his colleagues defined key terms such as “maximum O_2_ intake,” “O_2_ requirement,” and “steady state of exercise” (6), and his work on maximal oxygen uptake and oxygen debt became central to the later development of sport and exercise physiology (10). Hill's distinction between oxygen demand, oxygen uptake, and oxygen debt was especially important because it helped explain how intense exercise could proceed at rates exceeding the body's immediate aerobic capacity (42, 43). While later research revised the classical oxygen debt concept and replaced it with broader interpretations such as excess post-exercise oxygen consumption, Hill's formulation nevertheless provided an important early framework for understanding the metabolic consequences of high-intensity exercise (44, 45).

Hill also made a lasting contribution to muscle mechanics through the force–velocity relationship. His empirical equation was later known as the Hill equation (6, 46), as shown in Equation 1:

$$\begin{array}{c} (F+a)(v+b)=(F_{\max}+a)b \end{array}$$ (1)

This hyperbolic relation between force and shortening velocity became one of the classic formulations in muscle physiology and later informed analyzes of force–power characteristics in human movement (47). Recent reviews indicate that the lasting importance of Hill's mechanical work lies in the foundational framework it provided for later muscle modelling, particularly because Hill-type models offered a computationally tractable and conceptually accessible approach for biomechanical applications (48). Hill did not confine himself to laboratory muscle experiments. In 1925, Hill explicitly applied physiological reasoning to athletic performance by examining how far performance over different race distances could be understood in relation to underlying energetic principles (49). Furthermore, Hill recognized the significance of running economy, observing that performance could be limited not only by oxygen uptake and oxygen debt, but also by “clumsy and uneconomical movements” (6). However, later reviews have also emphasized that Hill-type models remain phenomenological simplifications rather than complete mechanistic accounts, and that their limitations become more evident in natural *in vivo* movement, especially under submaximal activation, rapid contractions, and in larger muscles (48, 50).

In this sense, Hill provided an early scientific framework for linking physiology with athletic performance and laid an essential foundation for later sprint research (10, 11, 39). However, Hill's framework remained primarily physiological and could not by itself provide the multidimensional mechanical explanation required in modern sprint science. Later sprint research increasingly considered metabolic factors alongside neuromuscular qualities, force application, and technical organization, thereby extending rather than replacing Hill's early physiological perspective (32). Accordingly, Hill's continuing relevance to sprint science lies less in offering a complete explanation of sprint performance than in establishing the physiological foundation upon which later biomechanical and integrative models were built.

### 3.2 Experience-Based training and the systematization of coaching

The development of sprint science was shaped not only by laboratory-based research but also by the gradual systematization of coaching practice. Before sprint training became closely connected with formal scientific analysis, much of its development depended on technical observation, experience-based judgment, and individualized training design. In this respect, Mussabini occupies an important place in the history of sprint coaching. Day emphasizes that coaches such as Mussabini worked through a mixture of explicit knowledge and tacit “craft” knowledge, relying on observation, intuition, and the practical “discerning eye” of the trainer rather than on academic science alone (34). Mussabini therefore represents an early form of systematic coaching, though not a fully science-led model in the modern sense (51, 52).

Mussabini was also interested in more systematic methods of performance evaluation. He was interested in the biomechanics of running and later used slow-motion film and photographic sequences to study athletes in action, examining stride length, arm swing, and phases of the sprint race (13, 51). Although his methods remained grounded primarily in practical judgment, technical observation, and individualized preparation, they also anticipated later attempts to make sprint coaching more structured and analytically informed (34, 52). Seen in this way, Mussabini's contribution lies not in providing a formal mechanistic explanation of sprinting, but rather in moving coaching practice toward a more systematic and analytically concept. Recent coaching scholarship has argued that historical and biographical analyses remain valuable because they show how coaching knowledge was transmitted, sustained, and adapted in practice (53). In this respect, Mussabini's continuing relevance lies in formalizing observational and experience-based coaching rather than in providing an explicit scientific theory of sprint performance.

Hart's coaching writings indicate that his method was systematic rather than *ad hoc*. He described the 400 m as an “endurance sprint” that combines the speed of a sprinter with the endurance of a half-miler, and argued that success depends on the efficient distribution of speed and energy across the race. Most importantly, he emphasized that the 400 m cannot be run “all out” from start to finish, making pace judgment and the efficient distribution of effort central coaching concerns (12). This practical insight still retains explanatory value, as recent modelling of world-class 400 m performance shows that athletes typically reach peak velocity early and then decelerate progressively, so performance depends largely on limiting the subsequent loss of velocity across the race rather than sustaining an maintaining a controlled fast pace (54).

A central feature of Hart's system was the planned organization of training across the year. He divided the season into four segments (off-season, early season, mid-season, and late season) and recommended different types of workouts, including speed endurance, tempo endurance, strength endurance, endurance running, power speed, event running, speed, and strength work. He also summarized his philosophy as moving from “quantity to quality,” while insisting that training should follow a progressive pattern and that coaches should be directly involved in pacing strategy and segment timing (12, 55). Studies on coaching planning have suggested that, although coaches continue to use seasonal structure and periodized phases, effective planning in practice is usually more flexible, holistic, and adaptive than the rigid linear models that are often associated with classical periodization (56). For the purposes of this mini review, Hart's significance lies primarily in the systematization of sprint training itself rather than in formal laboratory research. His work shows how coaching practice could become more structured, periodized, and analytically organized without losing its direct performance orientation. However, Hart's work remained focused primarily on training organization and applied coaching logic rather than on biomechanical quantification or mechanistic explanation. His importance therefore lies in showing how sprint coaching could be rationally structured in practice, even before later scientific models provided more explicit explanations of sprint performance.

Taken together, Mussabini and Hart illustrate how sprint coaching evolved from experience-based technical guidance toward a more systematic training framework. Their work is important not due to it provided a complete scientific account of sprint performance, but because it helped create a more organized practical foundation onto which later biomechanical and physiological analyses could be added.

### 3.3 The development of sprint biomechanics

As sprint research developed, biomechanical analysis increasingly became central to the scientific understanding of performance (7). Within this development, Mann and Hinrichs represent two related but distinct developments in sprint biomechanics: Mann integrated biomechanics into coaching practice, whereas Hinrichs broadened biomechanical understanding by highlighting the functional role of the upper extremities in running.

Mann contributed to sprint science by combining the perspective of an elite athlete with systematic biomechanical analysis. His early work helped establish sprinting as a subject that could be examined through measurable biomechanical variables rather than coaching observation alone (22, 57, 58). In particular, he emphasized the mechanics of the support phase, force management during touchdown and takeoff, and the contribution of lower-limb joint actions during ground contact (57, 59). His later Olympic analyses further strengthened the applied relevance of this approach by showing that elite sprint performance could be associated with variables such as higher horizontal velocity, greater stride rate, shorter support time, and more advantageous lower-limb mechanics (60, 61). Collectively, Mann's work provided an early biomechanical framework for interpreting sprint technique through quantitative indicators and brought biomechanical thinking closer to high-level coaching practice. However, this framework remained centered largely on technique description and observable mechanical variables. While highly valuable for coaching and training practice, it did not extend to a broader integrative account of sprint performance. Thus, Mann's contribution lay in rendering sprint technique more measurable and analytically interpretable, rather than in providing a complete explanatory model of sprint performance.

Hinrichs systematically focused on the upper extremities, a topic that had long received much less attention than the lower body in locomotion research (36, 62, 63). In his review of arm-leg coordination, he noted that locomotion research had often proceeded as if the arms and trunk were of little importance to the overall picture of gait (62). This point was already reflected in his earlier work with Cavanagh on treadmill walking, i.e., the movement of the arm center of mass was not simply a simple passive oscillation out of phase with the rest of the body (62, 64, 65). Subsequent work has generally reinforced this shift away from treating the upper extremities as passive appendages. Experimental and simulation studies indicate that arm swing primarily contributes to the control of whole-body rotation and torso stability, whereas its direct contribution to sprint speed appears comparatively modest (66).

Using three-dimensional cinematography, electromyography, and a 14-segment mathematical model, Hinrichs showed that arm action in running should not be regarded as simply auxiliary, but as part of whole-body coordination involving angular momentum regulation, movement stabilization, and coordination with the legs and trunk (20, 21, 64). His later case-study work on asymmetrical arm action further suggested that one arm could compensate for the other in generating angular momentum about the vertical axis, and that arm asymmetries could partially offset asymmetries elsewhere in the body, especially in the legs, thereby helping maintain overall balance of angular momentum about the vertical axis (67). Subsequent studies have refined rather than overturned this view. Restricting arm motion during short sprinting appears to compromise performance only marginally, but it increases the compensatory rotational demands placed on the torso, whereas active arm swing has been shown to reduce torso angular motion and lower the metabolic cost of running (36, 68, 69). Taken together, these studies expanded sprint biomechanics beyond a predominantly lower-limb focus and supported a more complete view of running as an integrated three-dimensional movement. However, Hinrichs' contribution addressed one important biomechanical dimension rather than providing a full performance model of sprinting. His work therefore broadened sprint biomechanics conceptually, but it functioned primarily as a corrective to lower-limb reductionism. Accordingly, the enduring influence of Hinrichs' work lies in its emphasis on angular-momentum regulation and whole-body coordination, together with the insight that arm action contributes only modestly to forward propulsion but makes a small yet significant contribution to lift. Subsequent research has largely reinforced this view, indicating that arm swing mainly serves to stabilize the trunk, reduce compensatory rotational demands, and improve running economy, while in the start and early acceleration phases it may also indirectly assist forward propulsion (36, 69).

### 3.4 Strength theory and Its relevance to sprint preparation

Zatsiorsky was not a sprint specialist in the narrow sense, but his work contributed importantly to the scientific foundations relevant to sprint preparation (24, 70–72). His place in the history of sprint science lies not primarily in direct sprint experimentation, but in providing concepts and methods that helped connect locomotion biomechanics with strength and power training theory.

A key example is his 1978 study with Aleshinsky, which analyzed normal gait, sport walking, and sprint running through a branching 15-link kinematic chain of the human body (23). The study aimed to determine the forces and moments at the major joints during three-dimensional motion and emphasized analysis of the entire body rather than isolated segments. A related biomechanical contribution can be seen in his 1994 paper with Prilutsky on two-joint muscles, which showed that mechanical energy could be transferred between joints during locomotion: from distal to proximal joints in the shock-absorbing phase, and from proximal to distal joints in the push-off phase (73). These studies strengthened an integrated biomechanical understanding of running as a coordinated whole-body process rather than a collection of separate joint actions. An important aspect of Zatsiorsky's contribution was that his work encouraged locomotion to be analysed as a coordinated whole-body mechanical process, an approach that later sprint biomechanics could develop in more event-specific ways.

Zatsiorsky discussed the importance of exercise specificity, the limited time available for force development in fast movements, and the distinction between maximal strength and the force actually expressed in explosive actions. His concept of the explosive strength deficit was particularly relevant to sprint training because it highlighted that increases in maximal strength do not automatically transfer to high-speed performance unless athletes can express that strength rapidly (30). Subsequent literature has generally supported the importance of these ideas, but it has also clarified their limits more precisely. Later reviews and meta-analyses indicate that lower-body strength gains can transfer positively to sprint performance, yet this transfer is influenced by training status, program design, and the specificity of the exercises performed (1, 74). Later work on maximal power production has likewise emphasized that, in explosive movements, the short time available for force development is a major determinant of performance (75, 76). Although these ideas were not formulated as a sprint-specific theory, they became influential in the design of sprint and other power-oriented training programs. Zatsiorsky's contribution lies in showing how strength theory could be connected to locomotion mechanics and to the practical problem of transferring general strength into sprint-relevant explosive performance (24, 30). What remains especially valuable in Zatsiorsky's framework is its emphasis that maximal strength and explosive performance should not be regarded as identical. Later research has also indirectly supported this view, as sprint performance depends not only on force magnitude but also on how rapidly and in what movement context force can be expressed (1, 77, 78).

Collectively, these contributions help explain why Zatsiorsky remains relevant to sprint science. Although he was not a sprint specialist in the narrow sense, his work helped connect integrated locomotion mechanics with later theories of strength transfer and explosive performance. However, because these concepts were not developed specifically for sprinting, their relevance to sprint science has been partly indirect and has depended on later adaptation, reinterpretation, and contextualization within sprint-specific literature and coaching practice. Seen from nowadays, Zatsiorsky's importance lies less in offering a complete sprint theory than in providing a conceptual bridge between mechanical coordination, strength qualities, and the practical problem of transferring general force capacity into sprint-specific performance.

### 3.5 The quantitative and model-based turn in sprint science

Weyand and his collaborators produced a body of highly cited research that advanced sprint biomechanics through the integration of mathematical and biomechanical modeling using a range of quantitative approaches such as regression models, differential equations, force-time analysis, and mechanical modeling. Its broader significance lies in improving explanation, prediction, and mechanistic interpretation in sprint performance research (8).

A key part of this quantitative turn was the force-based reinterpretation of top-speed running. Weyand and colleagues showed that faster top running speeds depend primarily on the application of greater ground reaction force rather than substantially faster leg movements in the air (27). This finding shifted quantitative sprint research toward a more force-centered interpretation of maximal-speed running. Subsequent work then moved from identifying force magnitude as important to examining more closely how these forces are generated during stance. In this respect, Clark and Weyand tested whether the fastest running speeds are achieved with the simple-spring stance mechanics predicted by the classic spring-mass model (28). Their findings suggested that they are not. Instead, the fastest runners deviated most from the simple-spring pattern, particularly at top speed, and applied greater vertical ground reaction force during the first half of stance (28). Taken together, these studies advanced sprint science from showing that force production is central to performance toward a more specific mechanical explanation of how force is applied within the brief stance period available in sprinting. Other work by Weyand also addressed performance prediction, structural interpretation, and the limits of running performance. His research addressed the limited effect of hypoxic reductions in aerobic power on high-speed running performance (79), high-speed running performance and prediction (80), the structural basis of running performance (81), sprint performance–duration relationships in relation to the fractional duration of external force application (82), and the biological limits of running speed (83). Considered together, these studies extended quantitative sprint research beyond a single empirical claim and toward a broader effort to explain how force production, gait structure, and performance limits could be analyzed within a mechanically interpretable framework.

Weyand's contribution lies not in any single empirical finding, but in the way his research helped move sprint science toward mathematically grounded explanations of force generation, gait mechanics, and performance limits. However, the explanatory scope of the early force-based framework remained limited, as later research suggested that sprint performance could not be fully explained by support-force magnitude alone, but also required more explicit consideration of stance mechanics, force-application timing, and the mechanical effectiveness with which force is oriented and applied during running (38, 84).

### 3.6 Integrative discussion across thematic strands

Analyzing these thematic strands reveals that the maturation of sprint science did not occur in isolation or along a singular linear progression. The foundational mapping of physiological bioenergetics, the development of applied coaching paradigms, the emergence of advanced biomechanical and kinetic analyses, and the formulation of quantitative strength theories collectively demonstrate a continuous cross-pollination of ideas. Primary advances in sprint performance emerged through the interaction of these complementary domains rather than through a single disciplinary pathway.

More specifically, physiology and biomechanics contributed at different but related explanatory levels. Physiological research helped define the energetic and muscular conditions under which sprinting is performed, whereas biomechanics clarified how movement, force generation, and application are expressed mechanically during sprint performance. In this sense, biomechanics did not replace physiology; rather, it extended sprint science by providing a more explicit account of how performance is mechanically realized.

A similar relationship can be seen between coaching practice and later scientific modeling. Early coaching traditions, as represented by Mussabini and Hart, did not provide formal mechanic explanations of sprint performance, but they played a crucial role in organizing the practical problems of training, pacing, and technique. Later scientific approaches in biomechanics and quantitative modeling made these practical concerns more measurable, analyzable, and open to verification. Thus, scientific modeling can be understood not as a rejection of coaching knowledge, but as a development that increasingly quantified and interpreted problems first recognized in practice.

Strength theory occupied an important bridging position within this broader development. In this respect, Zatsiorsky's work is especially significant because it linked general capacity development with sprint-specific performance requirements. By addressing issues such as specificity, explosive strength, and the transfer of force into rapid movement, strength theory helped connect physiological potential, training design, and biomechanical expression. It therefore served as an important conceptual bridge between the development of general athletic qualities and their realization in sprint mechanics and performance.

Overall, these thematic strands show that modern sprint science emerged through the interaction of energetic explanation, practical coaching organization, biomechanical interpretation, strength transfer theory, and quantitative modeling. Physiology helped define the energetic conditions of sprinting, coaching practice organized how performance was developed in training, biomechanics clarified how movement and force were expressed, strength theory addressed the transfer of general capacity into sprint-relevant output, and quantitative modeling increasingly linked these domains through prediction and mechanic interpretation. The historical development of sprint science was therefore fundamentally integrative, rather than reducible to any single disciplinary pathway.

## 4 Limitations and outlook

This mini review has several limitations. Due to the inherent brevity of the format, the selection of key figures is not exhaustive; rather, these individuals were chosen as representative lenses to illustrate broad thematic developments. Consequently, many other highly influential scientists and coaches who have significantly advanced sprint science could not be included. Furthermore, the focus on specific historical paradigms may inadvertently underrepresent contributions from diverse global sporting systems. In addition, because this review adopts a narrative and historically selective approach rather than a formal systematic design, the interpretation of intellectual influence and thematic priority inevitably involves a degree of author judgment. The use of key figures also risks simplifying the internal diversity of sprint science by grouping complex contributions into relatively concise thematic categories.

Future comprehensive systematic reviews and historical analyses are encouraged to expand upon this framework by incorporating a wider array of international researchers and alternative methodological approaches. At the same time, research on sprint training remains highly dependent on traditional biomechanical and theoretical training approaches, whereas broader AI applications in sport have developed more rapidly in adjacent areas of sports analytics, including computer vision, sensor-based systems, and data-driven performance analysis, with limited effective intersection between the two (85). Recent studies nevertheless suggest a possible pathway for narrowing this gap. In sprint and athletics-related contexts, deep learning has already been used to support technique and tactic evaluation through automated feature extraction from training and competition data, while newer movement-analysis frameworks have shown that athlete movements can be modelled effectively through integrated spatial–temporal representations (86–88). Studies in running and lower-extremity movement further indicate that, although these methods are promising, their current application still depends on sufficient accuracy, robustness, and task-specific validation (89, 90). Moreover, broader reviews of AI in sports performance analysis suggest that these developments continue to face important practical limitations, including variability in data quality, insufficiently standardized validation protocols, limited cross-task and cross-sport generalizability, and the continuing lack of unified open datasets and evaluation standards (91, 92). Future research may therefore usefully explore several new directions, including the use of computer vision to capture sprint technical parameters in real time, the application of reinforcement learning to generate individualized resistance- or slope-based training prescriptions, and the integration of dynamic systems theory with AI-based modeling to examine the evolution of individual coordination strategies. However, these directions should presently be regarded as promising research avenues rather than established applied solutions for sprint training.

## 5 Conclusion

The historical development of sprint science is best understood as a complex, multidisciplinary convergence rather than a simple linear pathway. By tracing influential contributions across physiology, biomechanics, strength theory, and applied coaching practice, this mini review highlights how the dynamic integration of diverse knowledge domains has shaped the modern scientific framework of sprinting. Moving forward, the continued evolution of the field will require strengthening these interdisciplinary connections. Future progress in sprint science may increasingly depend on artificial intelligence methods that have already been widely applied in other fields, as these approaches may offer new possibilities for enhancing existing biomechanical and physiological analyses.
