# Supplementary material for: Pioneers and paradigms in sprint science: a thematic historical mini review
Source: Front Sports Act Living. 2026 May 18;8:1843352. doi: 10.3389/fspor.2026.1843352 (PMC13222953; doi:10.3389/fspor.2026.1843352)
Supplement: Supplementary file 1 [file Datasheet1.pdf]

Pioneers and Paradigms in Sprint Science:

A Thematic Historical Mini Review

Supplementary Material

Illustrative Documentary Basis for the Identification and Verification of Key Figures

To improve transparency in this structured narrative mini review, this appendix provides an illustrative documentary account of how key figures were identified and retained for analysis. It was not intended to constitute a formal systematic review, a bibliometric ranking, or an exhaustive list of all contributors to sprint science. Rather, its purpose is to show, through selected examples, how broader thematic searches led to the identification of candidate figures, how these figures were then verified through targeted author-based searches, and how representative publications were retained for historical and thematic analysis. All searches reported in this appendix were conducted between 15 March 2026 and 18 March 2026, with final record verification on 20 April 2026.

1 General approach

Following the broader thematic and field-based screening described in the Methods, candidate figures were first identified when their work appeared repeatedly in connection with a major domain in sprint science, such as physiology, biomechanics, coaching methodology, strength theory, or quantitative analysis. At this stage, identification was provisional rather than final. The aim was not to extract names mechanically from keyword results, but to determine whether a given figure appeared repeatedly as a historically or conceptually important contributor within a relevant thematic strand.

Once a candidate figure had been identified provisionally, targeted author-based searches were conducted in order to verify that figure's relevance to sprint science and to identify representative publications for subsequent analysis. Publications were retained when they were judged to be historically influential, conceptually important, or especially useful for documenting the figure's contribution to the thematic development of sprint science. Citation prominence was considered only as contextual evidence of influence and not as a stand-alone inclusion criterion.

2 Illustrative example: Hill AV and the physiological strand

Within the broader thematic screening, searches using combinations of terms such as "exercise physiology," "sprint," "running," "muscle energetics," and related historical literature repeatedly led to foundational work on muscular work, oxygen uptake, and the physiological interpretation of athletic performance. Within this thematic strand, Hill emerged as a recurrent and historically important figure because his work appeared repeatedly in connection with the physiological foundations later used to interpret high-intensity exercise and sprint performance.

Hill was therefore identified provisionally as a candidate figure within the physiological strand. His relevance was then examined more directly through targeted author-based searches in Google

Scholar, PubMed where applicable, and historical scientific records using “Hill AV” together with terms such as “muscle,” “dynamic constants,” “force-velocity,” “exercise physiology,” “running” or “sprint.” Because Hill was not a sprint specialist in the narrow bibliographic sense, his inclusion was not based on the frequency of sprint-specific records alone. Rather, it was based on the fact that his work provided a physiological framework that later became important for the scientific interpretation of sprint performance.

This example illustrates the general logic used throughout the review: broader thematic searches first identified a relevant intellectual strand; repeated appearance and historical importance within that strand led to provisional identification of a candidate figure; targeted author-based searches were then used to verify relevance and retain representative publications; only after this process was the figure included for analysis in the main text.

3 Other key figures

The same general logic was applied to other retained figures, although the precise search route varied according to the nature of their contribution and documentary record.

For Mussabini SA and Hart C, broader thematic screening within the coaching and training strand used combinations of terms such as “sprint training,” “coaching,” “training methods,” “running,” “track and field,” “speed development,” and “athletic training.” These searches repeatedly led to historically important materials on the systematization of sprint coaching and training organization, through which Mussabini and Hart were identified provisionally as candidate figures. Because their contributions are preserved mainly through books, historical athletics materials, and coaching documents rather than through a large indexed journal record, subsequent verification relied more heavily on books, library catalogues, and coaching-oriented sources.

For Mann RV and Hinrichs RN, broader thematic screening within the biomechanics strand used combinations of terms such as “sprint biomechanics,” “running biomechanics,” “sprint running,” “kinetic analysis,” and “kinematic analysis.” These searches repeatedly identified work on sprint mechanics, running biomechanics, and whole-body coordination, through which Mann and Hinrichs were identified provisionally as candidate figures. They were then verified through targeted author-based searches using sprint- and running-related biomechanical terms.

For Zatsiorsky VM, broader thematic screening within the strength and integrated mechanics strand used combinations of terms such as “strength training,” “explosive strength,” “locomotion mechanics,” “human motion,” “biomechanics,” “running,” “kinetics,” and “movement analysis.” These searches repeatedly identified work relevant to locomotion mechanics, strength transfer, and the mechanical analysis of movement, through which Zatsiorsky was identified provisionally as a candidate figure. Because his contribution to sprint science is partly indirect and conceptual rather than based on a narrow sprint-only literature, subsequent verification relied on both journal articles and foundational biomechanics or strength-training texts.

For Weyand PG, broader thematic screening within the quantitative and force-based strand used combinations of terms such as “ground reaction force,” “running mechanics,” “sprint performance,” “maximal-speed running,” “force application,” “performance prediction,” and “biomechanics.” These searches repeatedly identified work on force-centered interpretations of sprinting and quantitative analyses of running performance, through which Weyand was identified provisionally as a candidate

figure. Targeted author-based searches then verified his relevance and helped retain a body of representative sprint-related publications.

4 Representative publications retained for documentary support

The following works were retained as representative documentary anchors for the selected figures:

4.1 Hill AV

Hill AV. The maximum work and mechanical efficiency of human muscles, and their most economical speed. *J. Physiol.* (1922) 56(1–2):19–41. doi: 10.1113/jphysiol.1922.sp001989

Hill AV. The heat of shortening and the dynamic constants of muscle. *Proc. Biol. Sci.* (1938) 126(843):136–195. doi: 10.1098/rspb.1938.0050

Hill AV. The physiological basis of athletic records. *Sci. Mon.* (1925) 21(4):409–428.

4.2 Mussabini SA

Mussabini SA. *The complete athletic trainer*. London: Methuen & Co. (1913). 330 p.

Day D. Craft coaching and the 'discerning eye' of the coach. *Int. J. Sports Sci. Coach.* (2011) 6(1):179–195. doi: 10.1260/1747-9541.6.1.179

Day D. Bricoleurs extraordinaire: sports coaches in inter war britain. *Sports Coach. Rev.* (2019) 8(3):243–261. doi: 10.1080/21640629.2018.1521624

Carter N. From Knox to Dyson: coaching, amateurism and British athletics, 1912–1947. *Sport Hist.* (2010) 30(1):55–81. doi: 10.1080/17460261003616716

Day D. 'Magical and fanciful theories': sports psychologists and craft coaches. *Sports Coach. Rev.* (2012) 1(1):52–66. doi: 10.1080/21640629.2012.702468

Day D, Loudcher J-F, Vaucelle S. Sports coaching histories and biographies: a raison D'être. *Sports Coach. Rev.* (2025) 14(1):1. doi: 10.1080/21640629.2024.2321804

4.3 Hart C

Hart C. 400 meter training. *Track Field Q. Rev.* (1981) 93(1):23–28.

Lee J. *Clyde Hart training seminar notes*. SpeedEndurance.com (2007) https://speedendurance.com/2007/07/31/clyde-hart-training-seminar-notes/ [Accessed March 5, 2026]

4.4 Mann RV

Mann R. *The mechanics of sprinting and hurdling*. Charleston, SC, USA: CreateSpace Independent Publishing Platform. (2011). 206 p.

Mann R, Sprague P. A kinetic analysis of the ground leg during sprint running. *Res. Q. Exerc. Sport* (1980) 51(2):334–348. doi: 10.1080/02701367.1980.10605202

Sprague P, Mann RV. The effects of muscular fatigue on the kinetics of sprint running. *Res. Q. Exerc. Sport* (1983) 54(1):60–66. doi: 10.1080/02701367.1983.10605273

Mann RV. A kinetic analysis of sprinting. *Med. Sci. Sports Exerc.* (1981) 13(5):325–328. doi: 10.1249/00005768-198105000-00010

Mann R, Herman J. Kinematic analysis of Olympic hurdle performance: women's 100 meters. *J. Appl. Biomech.* (1985) 1(2):163–173. doi: 10.1123/ijsb.1.2.163

Mann R, Herman J. Kinematic analysis of Olympic sprint performance: men's 200 meters. *J. Appl. Biomech.* (1985) 1(2):151–162. doi: 10.1123/ijsb.1.2.151

4.5 Hinrichs RN

Hinrichs RN, Cavanagh PR, Williams KR. Upper extremity function in running. I: Center of mass and propulsion considerations. *J. Appl. Biomech.* (1987) 3(3):222–241. doi: 10.1123/ijsb.3.3.222

Hinrichs RN. Upper extremity function in running. II: Angular momentum considerations. *J. Appl. Biomech.* (1987) 3(3):242–263. doi: 10.1123/ijsb.3.3.242

Hinrichs RN. "Whole body movement: Coordination of arms and legs in walking and running.," In: Winters JM, Woo SL-Y, editors. *Multiple Muscle Systems: Biomechanics and Movement Organization.* New York: Springer (1990). p. 694–705. doi: 10.1007/978-1-4613-9030-5_45

Hinrichs RN. *Upper extremity function in running.* [dissertation]. University Park, PA: Pennsylvania State University (1982). Available at: https://search.proquest.com/openview/4470c9078e513c02cedcdbf2ecc7bc0d/1?pq-origsite=gscholar&cbl=18750&diss=y (Accessed June 13, 2025)

Hinrichs RN. Case studies of asymmetrical arm action in running. *Int. J. Sport Biomech.* (1992) 8:111–128. doi: 10.1123/ijsb.8.2.111

4.6 Zatsiorsky VM

Aleshinsky SY, Zatsiorsky VM. Human locomotion in space analyzed biomechanically through a multi-link chain model. *J. Biomech.* (1978) 11(3):101–108. doi: 10.1016/0021-9290(78)90002-7

Zatsiorsky VM, Kraemer WJ, Fry AC. *Science and practice of strength training*. Champaign: Human Kinetics. (2020). 346 p.

Zatsiorsky VM. *Kinetics of human motion*. Champaign: Human Kinetics. (2002). 680 p.

Zatsiorsky V. *Biomechanics in sport: performance enhancement and injury prevention*. Oxford: Blackwell Science Ltd. (2000). 683 p.

Prilutsky BI, Zatsiorsky VM. Tendon action of two-joint muscles: transfer of mechanical energy between joints during jumping, landing, and running. *J. Biomech.* (1994) 27(1):25–34. doi: 10.1016/0021-9290(94)90029-9

4.7 Weyand PG

Weyand PG, Sternlight DB, Bellizzi MJ, Wright S. Faster top running speeds are achieved with greater ground forces not more rapid leg movements. *J. Appl. Physiol.* (2000) 89(5):1991–1999. doi: 10.1152/jappl.2000.89.5.1991

Clark KP, Weyand PG. Are running speeds maximized with simple-spring stance mechanics? *J. Appl. Physiol.* (2014) 117(6):604–615. doi: 10.1152/japplphysiol.00174.2014

Clark KP, Ryan LJ, Weyand PG. A general relationship links gait mechanics and running ground reaction forces. *J. Exp. Biol.* (2017) 220(Pt 2):247–258. doi: 10.1242/jeb.138057

Weyand PG, Lee CS, Martinez-Ruiz R, Bundle MW, Bellizzi MJ, Wright S. High-speed running performance is largely unaffected by hypoxic reductions in aerobic power. *J. Appl. Physiol.* (1999) 86(6):2059–2064. doi: 10.1152/jappl.1999.86.6.2059

Bundle MW, Hoyt RW, Weyand PG. High-speed running performance: a new approach to assessment and prediction. *J. Appl. Physiol.* (2003) 95(5):1955–1962. doi: 10.1152/japplphysiol.00921.2002

Weyand PG, Davis JA. Running performance has a structural basis. *J. Exp. Biol.* (2005) 208(Pt 14):2625–2631. doi: 10.1242/jeb.01609

Weyand PG, Lin JE, Bundle MW. Sprint performance-duration relationships are set by the fractional duration of external force application. *Am. J. Physiol., Regul. Integr. Comp. Physiol.* (2006) 290(3):R758–765. doi: 10.1152/ajpregu.00562.2005

Weyand PG, Sandell RF, Prime DNL, Bundle MW. The biological limits to running speed are imposed from the ground up. *J. Appl. Physiol.* (2010) 108(4):950–961. doi: 10.1152/japplphysiol.00947.2009
